# Injection-based hairy root induction and plant regeneration techniques in Brassicaceae

**DOI:** 10.1186/s13007-024-01150-1

**Published:** 2024-02-17

**Authors:** Veronika Jedličková, Marie Štefková, Terezie Mandáková, Juan Francisco Sánchez López, Marek Sedláček, Martin A. Lysak, Hélène S. Robert

**Affiliations:** 1 Mendel Center for Plant Genomics and Proteomics, Central European Institute of Technology, Masaryk University, Brno, Czech Republic; 2 Department of Experimental Biology, Faculty of Science, Masaryk University, Brno, Czech Republic; 3 National Centre for Biomolecular Research, Faculty of Science, Masaryk University, Brno, Czech Republic

**Keywords:** Hairy root, Crucifers, Brassicaceae, *Asperuginoides axillaris*, *Arabidopsis thaliana*, *Cardamine hirsuta*, Transformation, Plant regeneration, Cytogenetics

## Abstract

**Background:**

Hairy roots constitute a valuable tissue culture system for species that are difficult to propagate through conventional seed-based methods. Moreover, the generation of transgenic plants derived from hairy roots can be facilitated by employing carefully designed hormone-containing media.

**Results:**

We initiated hairy root formation in the rare crucifer species *Asperuginoides axillaris* via an injection-based protocol using the *Agrobacterium* strain C58C1 harboring a hairy root-inducing (*Ri*) plasmid and successfully regenerated plants from established hairy root lines. Our study confirms the genetic stability of both hairy roots and their derived regenerants and highlights their utility as a permanent source of mitotic chromosomes for cytogenetic investigations. Additionally, we have developed an effective embryo rescue protocol to circumvent seed dormancy issues in *A. axillaris* seeds. By using inflorescence primary stems of *Arabidopsis thaliana* and *Cardamine hirsuta* as starting material, we also established hairy root lines that were subsequently used for regeneration studies.

**Conclusion:**

We developed efficient hairy root transformation and regeneration protocols for various crucifers, namely *A. axillaris*, *A. thaliana*, and *C. hirsuta*. Hairy roots and derived regenerants can serve as a continuous source of plant material for molecular and cytogenetic analyses.

**Supplementary Information:**

The online version contains supplementary material available at 10.1186/s13007-024-01150-1.

## Background

Brassicaceae is an economically important family of flowering plants, also known as the mustard family or crucifers. The genus *Brassica* includes some of the most important vegetable and oilseed crops grown worldwide, with diploid and allotetraploid species [1]. A tiny diploid crucifer species, *Arabidopsis thaliana*, has been adopted as a model species for the study of molecular, cellular, and developmental mechanisms in plants due to its rapid growth, self-fertilization, and small genome size [2].

The *A. thaliana* genome sequence and dozens of reconstructed karyotypes and chromosome-scale genome assemblies have established the Brassicaceae as a model family to study key mechanisms of plant genome evolution, including the processes of domestication and crop diversification. Plant material from different organs and developmental stages is needed for all cytogenetic and genomic studies. However, for some species in the Brassicaceae and other plant families, seed supply may be limited (e.g., in endangered or rare species), seeds may be difficult to germinate, plants may not be readily maintained in culture, and repeated collection of plant material in the wild may be impractical or even impossible. In addition, the short stature of plants or the small size of their organs may hamper DNA/RNA isolation and identification of meristematic or meiotic tissue for chromosome preparation. To circumvent these obstacles, in vitro cultures of the plant species under study can be established. Such cultures allow plants to be grown in a sterile, controlled environment and produce a large number of plants from a small amount of original plant material. In vitro cultures can be used for the conservation of rare or endangered plant species [3–5] and the production of disease-free plants.

Hairy root culture is a plant tissue culture widely used in plant biotechnology. Hairy roots are derived from the infection of a host plant with *Agrobacterium* strains harboring a root-inducing (*Ri*) plasmid. After infection, a T-DNA fragment of the *Ri* plasmid is transferred to plant cells and integrated into the genome. Expression of T-DNA-encoded genes, especially *root oncogenic loci* (*rol*) genes, enables the development of hairy roots [6–8]. In addition to using hairy root cultures as a tool to produce valuable secondary metabolites, transformed roots have been used for gene functional analyses, production of recombinant proteins, or genome editing using Clustered Regularly Interspaced Short Palindromic Repeats (CRISPRs)/CRISPR-associated protein 9 (Cas9) by co-transforming the *Ri* plasmid with an artificial binary vector [9–11]. Hairy root technology has also been proposed for the conservation of rare, endangered, or endemic species [9].

After developing an optimized protocol for hairy root transformation in *Brassica napus* [12], we tested the method of injection-based induction of hairy roots and subsequent regeneration of plants from hairy root cultures in different crucifer species. Here, we used hairy root cultures to obtain aseptic plants of *Asperuginoides axillaris*, a rare crucifer species of the Irano-Turanian floristic region (localities from Turkey to Kyrgyzstan, [13]). This rare species has a delicate leaf morphology and small single flowers in the leaf axils, which contain very few pollen mother cells, making efficient preparation of meiotic chromosome spreads difficult. In addition, we developed an embryo rescue protocol for *A. axillaris* to bypass the maturation phase of seed development and increase the yield of aseptic plants. We also show that the inflorescence primary stem is suitable for the induction of hairy roots in two model crucifers, Arabidopsis and *Cardamine hirsuta* (hairy bittercress).

## Results

### Exploration of cross-genera applicability of hairy root induction protocol

**Fig. 1:**
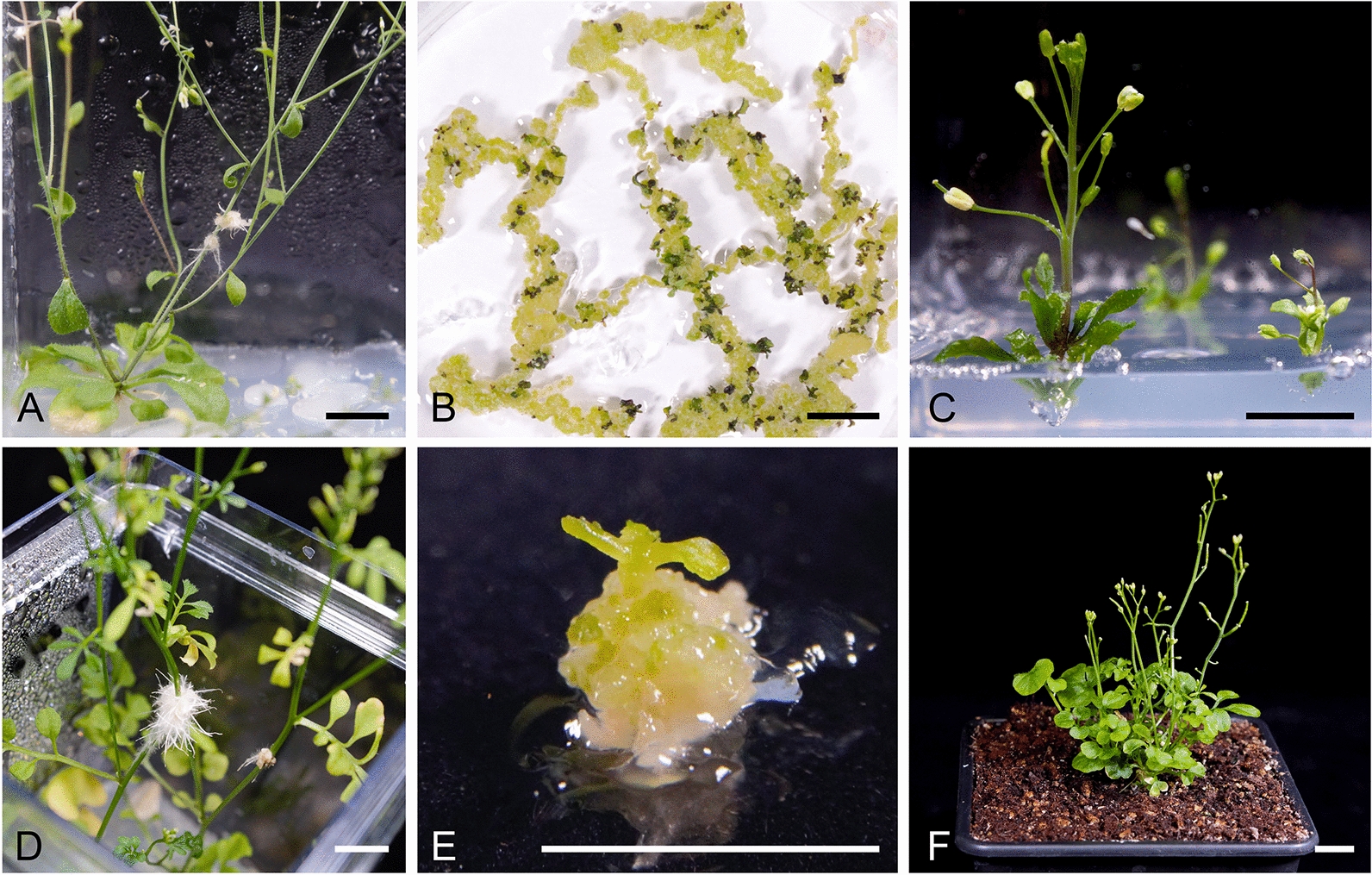
Hairy root induction and regeneration in *A. thaliana* and *C. hirsuta*. Hairy roots emerging from the site of inoculation in *A. thaliana* **A** and *C. hirsuta* **D**. Shoot induction on regeneration media in *A. thaliana* **B** and *C. hirsuta* **E**. **C,** Early flowering of *A. thaliana* regenerants on shoot elongation media. **F**, Regenerant of *C. hirsuta* in soil. Scale bars represent 1 cm

Previously, we successfully optimized a protocol for inducing hairy roots by injection and subsequent shoot regeneration in the *B. napus* cultivar DH12075 [12]. To assess the adaptability of this protocol to other genera in the Brassicaceae family, we initiated our investigation with the well-established model species *A. thaliana*. Given the distinct rosette growth of *A. thaliana* in contrast to the long hypocotyl of *B. napus*, we directed our injections to the inflorescence primary stem. One-month-old *A. thaliana* plantlets were injected at the base of the inflorescence stem using an *Agrobacterium* suspension containing the virulent *Ri* plasmid. Remarkably, transformation efficiency, quantified as the ratio of plants with emerging hairy roots at the inoculation site to the total number of injected plants, averaged 94%. This calculation was based on data collected one month after injection from three independent transformation experiments with 10 to 12 plants each (Fig. 1A). When we extended the age of *A. thaliana* plants to six weeks, transformation efficiency decreased slightly to 75 ± 8%. Hairy roots obtained from *A. thaliana* were carefully excised and cultivated in Petri dishes on solid Murashige and Skoog medium supplemented with B5 vitamins (MS + B5).

To initiate the formation of shoots, we transferred ten independent hairy root lines to a regeneration medium (RGM). The RGM contained auxin 1-naphthaleneacetic acid (NAA) at a concentration of 8 mg/L and the cytokinin 6-benzylaminopurine (BAP, 5 mg/L). Calli development was observed in all ten lines, and shoots formed in nine of the ten lines cultured (indicating a robust regeneration efficiency of 90%). This regeneration efficiency was similar to that achieved with *B. napus* DH12075 (Table 1). The emergence of the first shoots was evident after a cultivation period of 18 to 21 days (Fig. 1B). Subsequently, these shoots were excised and relocated to a shoot elongation medium (SEM), where they were nurtured for 2 to 3 weeks to promote further growth and elongation. Following this phase, the shoots were transferred to the root induction medium (RIM). The resulting rooted plants were then transplanted into the soil for further development. The hairy root regenerants exhibited distinct phenotypic characteristics, such as dwarf stature and a dense root system. These regenerants initiated flowering while still on the SEM, even before the emergence of roots. This transition to flowering occurred within only 40 days of culture (Fig. 1C). Such morphological alterations are consistent with well-documented observations in hairy root-derived plants, a phenomenon recognized as the hairy root or *Ri* phenotype [6].

**Table 1:** Hairy root induction using the injection-based method in various Brassicaceae species

Species	Transformation efficiency [%]	Regeneration efficiency [%]	References
*Arabidopsis thaliana* Col-0	94 ± 5	90	This study
*Asperuginoides axillaris*	33*	29	This study
*Brassica napus*			
DH12075	97 ± 3	90	[12]
Topas DH4079	42 ± 4	n.d	[12]
Westar	84 ± 13	n.d	[12]
*Cardamine hirsuta*	93 ± 6	30	This study

Transformation efficiency was calculated one month after the injection from 3 independent transformation assays, 10–12 plants per assay. Data are presented as mean ± standard deviation.  * In wild-type *A. axillaris*, three plants were subjected to transformation. Regeneration efficiency was tested on ten hairy root lines except for *A. axillaris*, where 14 lines were tested

To demonstrate the broad applicability of inflorescence primary stems as a valuable substrate for hairy root induction by injection within the Brassicaceae family, we conducted an experiment with another species characterized by a rosette-like growth habit, *Cadamine hirsuta*. One-month-old plantlets of *C. hirsuta* were subjected to injections at the base of the inflorescence primary stem. Approximately one month after the transformation, we observed the emergence of hairy roots in an average of 93% of treated plants (Fig. 1D). Subsequent analysis of hairy root lines of *C. hirsuta* revealed a regeneration efficiency of 30% (Table 1, Fig. 1E, F). These results highlight the suitability of the inflorescence stem as a highly efficient platform for hairy root induction in both model species. Moreover, the protocol used for shoot induction using auxin NAA and cytokinin BAP proved to be functional in both species.

### Hairy roots and regenerants in *A. axillaris*

The seeds of *A. axillaris* were collected in Semnan province of Iran. To establish an aseptic culture of *A. axillaris* plants, seeds were subjected to a sterilization process with ethanol treatment. Subsequent cultivation on germination medium 1 (GM1) consisting of MS medium containing 0.25 mg/mL gibberellic acid (GA_3_) resulted in remarkably low germination efficiency (3.3%, Additional File 1: Table S1). To improve this germination rate, seeds were sterilized with ethanol and treated with sodium hypochlorite solution. Treated seeds were cultivated on a modified MS medium, supplemented with B5 vitamins, 0.4% activated charcoal, and 0.1 mg/mL GA_3_ (Germination Medium 2 or GM2). This modified approach resulted in a slight improvement in germination rate, reaching 4.6%. Noteworthily, sterilization of the seeds with chlorine gas proved inefficient as it resulted in microbial contamination, rendering all seeds non-viable.

**Fig. 2:**
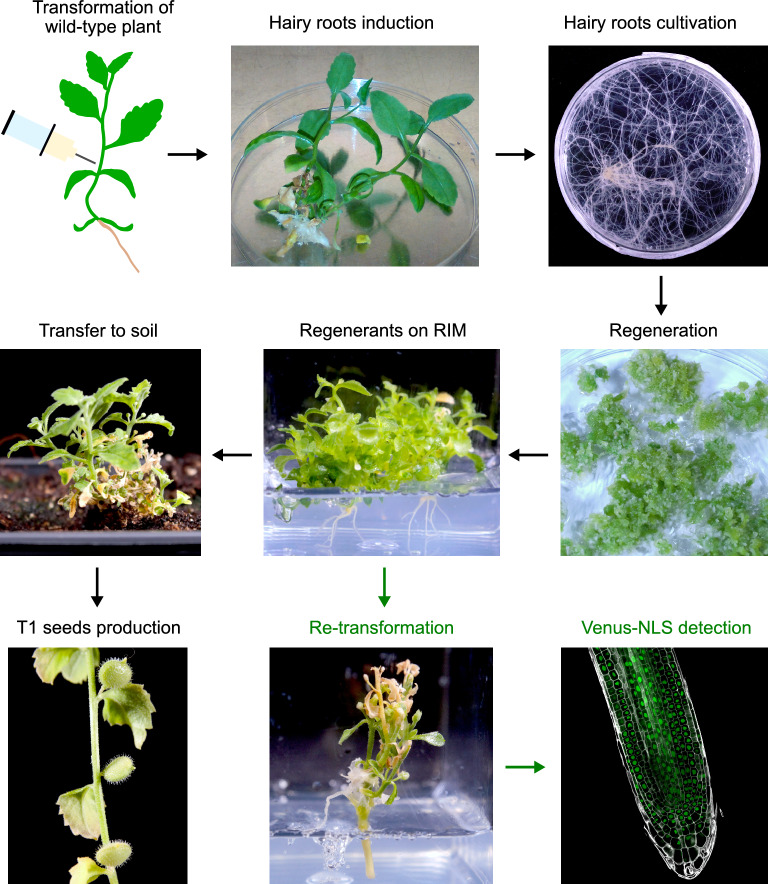
Process of hairy root induction and regeneration in *A. axillaris*. Hairy roots were induced by injection with *Agrobacterium* carrying a *Ri* plasmid. Selected hairy root lines were cultivated on Petri dishes. The shoot formation was elicited by culturing hairy roots on the regeneration medium. The shoots were subsequently transferred to the shoot elongation medium (SEM) and the root induction medium (RIM). Rooted plants were transferred to the soil and produced T1 hairy root-derived seeds. Alternatively, regenerants on RIM were subjected to another injection-based transformation with *Agrobacterium* carrying both the *Ri* plasmid and a binary vector expressing a nuclear-targeted fluorescent protein VENUS (VENUS-NLS) and carrying a kanamycin resistance cassette. Emerging hairy root lines were selected on kanamycin and screened for fluorescent signal

Three plants cultivated in aseptic conditions were subjected to injection with an agrobacterial suspension. Hairy roots emerged from one of the injected plants two months after transformation (33% transformation efficiency, Table 1, Fig. 2). Fourteen independent hairy root lines were established (Fig. 2), and successful transformation was monitored by genotyping the insertion of TL and TR from the *Ri* plasmid into the *A. axillaris* genome. The absence of agrobacteria contamination was confirmed by the absence of the *virC* locus in the extracted DNA (Additional File 1: Figure S1).

*A. axillaris* hairy root lines were cultivated on the RGM to induce shooting. Within one month, all 14 lines created yellow or green calli. In four lines (29%), the shoots emerged after 2–3 months of culture (Fig. 2). Hairy root-derived regenerants (T0 plants) were morphologically distinct from wild-type plants, having shortened internodes and smaller leaves (Fig. 2, Additional File 1: Figure S2). Regenerants flowered earlier than wild-type plants. Indeed, flower buds were detected on RIM before transfer to soil. Regions of T-DNA from the *Ri* plasmid (TL and TR) were detected in the genome of regenerants from all four lines (leaves from 3 to 5 regenerants per line were tested). One of the four lines did not set viable seeds, as the developed siliculas were empty (Additional File 1: Table S2).

### Re-transformation of *A. axillaris* regenerants

We tested the possibility of re-transformation, i.e., induction of hairy roots in regenerating *A. axillaris* plantlets growing on RIM. Regenerants were injected with a suspension of an agrobacterial strain carrying both the *Ri* plasmid and a binary vector expressing the fluorescent protein VENUS targeted to the nucleus. Within 1 – 2 months, hairy roots emerged from a stem of T0 regenerants (Fig. 2). The efficiency of the re-transformation was 29 ± 4%, resembling the transformation efficiency of *A. axillaris* wild-type plants. Hairy roots grown on a medium containing a selective agent (kanamycin, 15 mg/L) exhibited fluorescence (Fig. 2). The method of re-transformation is of great importance when seeds are sterile or non-viable.

### Germination of *A. axillaris* T1 seeds

The germination rate of hairy root-derived T1 seeds sown directly in soil or surface-sterilized with ethanol and sodium hypochlorite solution and aseptically sown did not exceed 5% (Additional File 1: Table S1). Hence, different treatments were tested to break seed dormancy.

Germination studies on *A. thaliana* Cvi showed that seed dormancy could be broken by treatment with cold or nitrate followed by light exposure. The sensitivity of seeds to such treatments depends on the storage period of dry and ripe seeds after harvest [14]. Therefore, all tested seeds spent at least six months in dry storage after ripening/harvesting. As no endogenous contamination was observed in T1 seeds, we used a sterilization procedure with chlorine gas. Moreover, dry siliculas were removed manually before sterilization. To overcome seed dormancy, we tested the effect of cold stratification at 4 °C in the dark on a solid GM3 (0.4% charcoal, 0.5 mg/L GA_3_, and 0.1% Plant Preserve Mixture) and imbibition of seeds in solution with high concentrations of GA_3_ (1 mg/mL) and KNO_3_ (0.5%) on filter paper (Additional File 1: Table S1). After the treatment, seeds were cultivated at 21 °C under a 16=h light/8-h dark photoperiod on GM3. A combination of 24 h imbibition in GA_3_ + KNO_3_ solution and cold stratification for two weeks increased the germination rate to 35% (Additional File 1: Table S1).

### Embryo rescue of immature *A. axillaris* T1 seeds

**Fig. 3:**
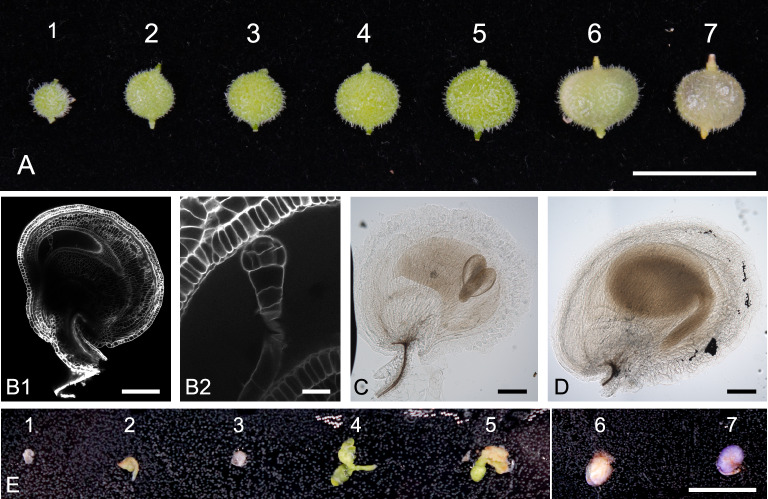
Embryo rescue in *A. axillaris* hairy root-derived seeds. **A**, Seven developmental stages of seeds based on size and maturation. **B1**, Seeds at stage 2 contain a globular embryo. **B2**, Higher magnification of B1, zoomed on the embryo. **C,** A torpedo embryo was detected in stage 5. **D**, A mature embryo was observed in seeds at stage 6. **E**, Seeds at stages 4–5 germinated on embryo rescue medium, whereas seeds at earlier or older stages did not germinate. Scale bars represent 1 cm (**A**, **E**), 1 mm (**B1**, **C**, **D**), and 100 µm (**B2**)

We tested the embryo rescue (ER) technique in *A. axillaris* seeds to bypass seed dormancy. Because the effective embryonic stage for ER in *B. napus* was the torpedo stage [12], we first identified the embryonic stages in seeds of different maturation ages. We defined seven seed stages according to their size and color: stage 1 – 2.8 ± 0.2 mm in diameter; stage 2 – 3.9 ± 0.2 mm; stage 3 – 4.4 ± 0.2 mm; stage 4 – 5.1 ± 0.2 mm; stage 5 – 5.6 ± 0.2 mm; stage 6 – 6.5 ± 0.2 mm; stage 7 – 5.6 ± 0.2 mm. Seeds of stages 1–5 were green, whereas those of stages 6 and 7 were yellow. Stage 2 seeds contained globular embryos (Fig. 3B1, B2). Torpedo embryos were detected in stage 5 seeds (Fig. 3C), while mature embryos were observed in stage 6 seeds (Fig. 3D). To confirm the suitability of torpedo embryos for ER experiments in *A. axillaris*, seed testa of all stages was opened under sterile conditions, and the seeds were cultivated on embryo rescue medium 4 (ERM4, Table 2). Indeed, the seeds of stages 4 – 5 germinated, whereas the younger and older seeds did not (Fig. 3E). We successfully induced ER in seeds of plants derived from all three hairy root lines capable of producing seeds (Additional File 1: Table S2). In addition, ER was also induced in T2 seeds of previously embryo-rescued T1 plants.

**Table 2:** Embryo rescue in *A. axillaris* hairy root-derived T1 seeds

ER media	GA_3_ [mg/L]	BAP [mg/L]	IAA [mg/L]	Charcoal [%]	Germination rate [%]
ERM1	0.5	–	–	0.4	39 ± 3
ERM2	0.5	1	–	0.4	41 ± 5
ERM3	0.5	–	1	0.4	42 ± 5
ERM4	0.5	1	1	0.4	66 ± 4

Growing media	GA_3_ [mg/L]	BAP [mg/L]	IBA [mg/L]	Survival rate [%] (n)	
MS + B5	–	–	–	9 (2/23)	
SEM	0.03	0.5	–	53 (18/34)	
RIM	–	–	0.5	37 (14/38)	

3–4 independent ER assays were performed with 11–14 seeds per assay in different embryo-rescue media (ERM). For details, see Methods and Results. The germinating seeds were transferred to cultivation boxes with either medium without hormone, shoot elongation medium (SEM), or root inducing medium (RIM). The survival rate was calculated two months after the transfer

We also tested the efficacy of ER in seeds (stage 4–5) germinated on cultivation media containing different combinations of gibberellic acid (GA_3_), auxin (indole-3-acetic acid, IAA), and cytokinin (BAP) (ERM1–4, Table 2). The highest germination rate was observed for the ERM4 medium containing all three hormones (66 ± 4%). The germinating seeds were transferred in equal proportions to SEM, RIM, or medium without hormones. The highest survival rate was observed for seeds transferred to SEM (counted two months after the transfer, Table 2). The surviving plantlets in all media formed roots and were subsequently transferred to soil.

Thus, the optimal protocol for ER in *A. axillaris* involves the use of torpedo-stage embryos (green seeds with a diameter of approximately 5.1 mm–5.6 mm) cultured on MS + B5 medium containing 0.5 mg/L GA_3_, 1 mg/L BAP, 1 mg/L IAA, and 0.4% charcoal and transferred after germination to cultivation boxes with SEM containing 0.5 mg/L of BAP and 0.03 mg/L of GA_3_.

### *A. axillaris* T1 plants

**Fig. 4:**
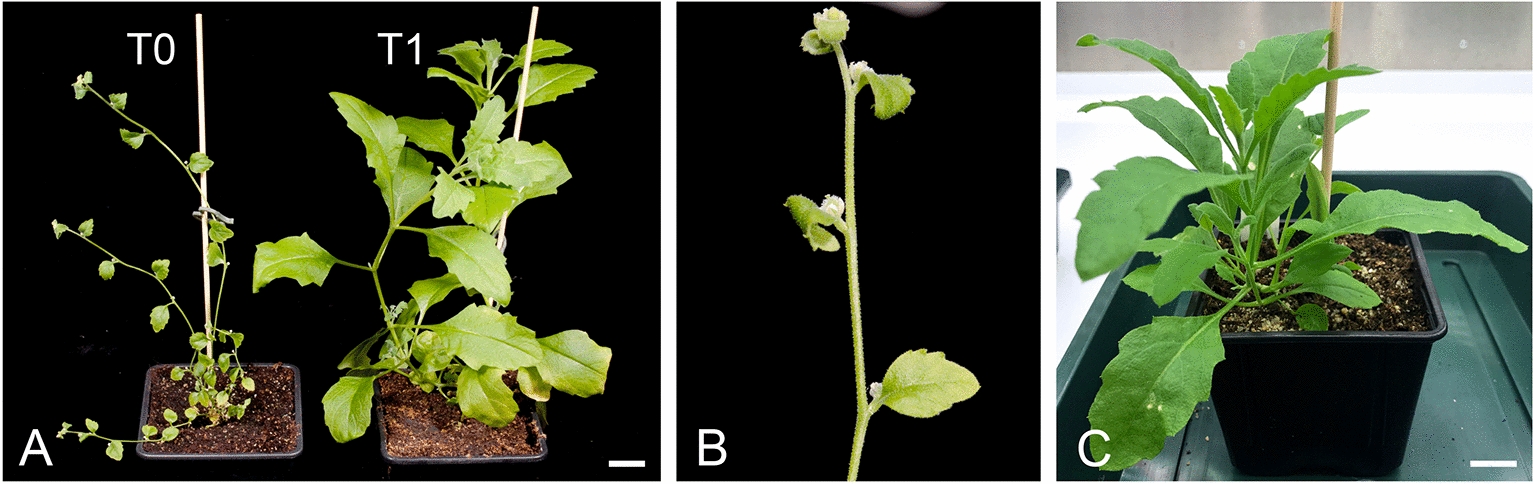
Comparison of *A. axillaris* hairy root-derived regenerant with T1 plant. **A**, Representative regenerant (T0 plant on the left) is compared to the T1 plant (on the right). **B**, Detail of developing seeds in T0 regenerant. No flowers or seeds are detected in T1 plants of the same stature. **C**, Wild-type *A. axillaris* plant. Scale bars represent 2 cm

T1 plants (8, 6, and 7 plants from Line 2, Line 5, and Line 7, respectively) transferred to soil were screened for the presence of the *Ri* T-DNA (TL and TR regions) in the genome. TL and TR regions were detected in genomic DNA extracted from leaves of all T1 plants derived from Line 7. All T1 plants derived from Line 2 and Line 5 contained the TL region, but some plants (1/8 and 2/6 of Lines 2 and 5, respectively) did not carry the TR region (Additional File 1: Table S2). Figure 4 shows a comparison of T0 regenerants and T1 plants. T1 plants lost their early flowering, characteristic of the T0 regenerants. They flowered several weeks after transfer to soil and resembled the wild-type phenotype (Fig. 4C). The loss of hairy root phenotype may occur by T-DNA gene silencing [6], which is supported by the detection of the *A. rhizogenes* T-DNA-derived small RNAs in hairy roots [15].

### Cytogenetic evaluation of hairy roots and regenerants of *A. axillaris* for chromosomal stability

**Fig. 5:**
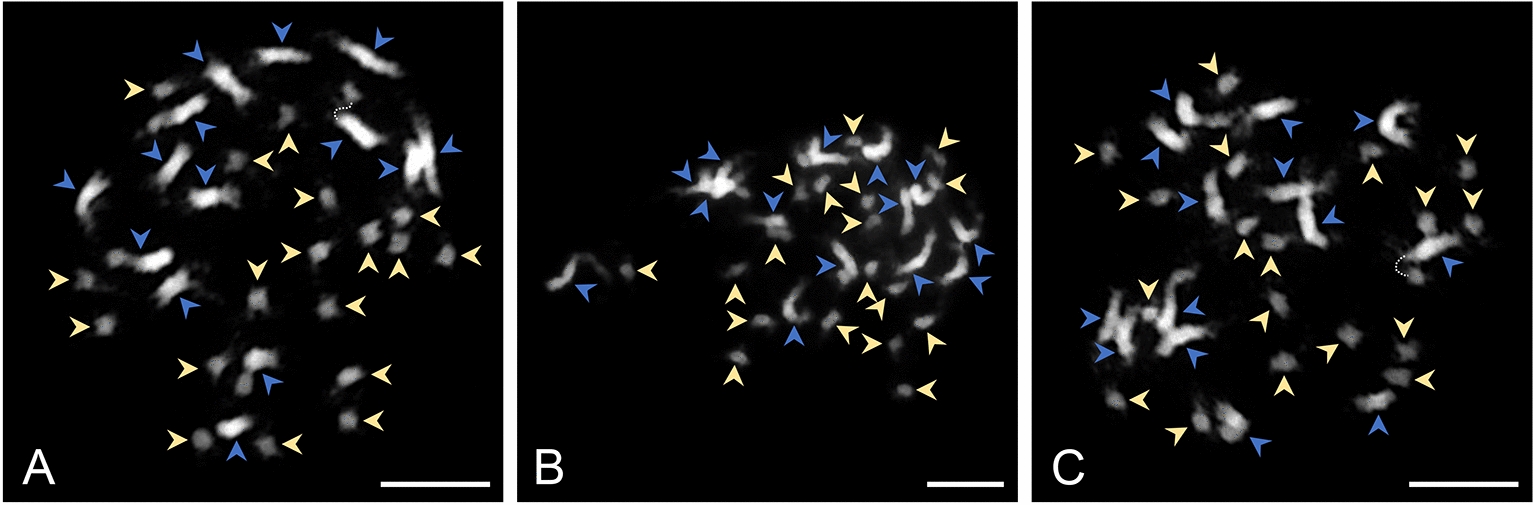
Mitotic chromosomes of *A. axillaris* prepared from root tips. **A**, Plant cultivated from seeds (wild type). **B**, Hairy roots. **C**, T0 regenerant. The karyotypes (2n = 32) display a bimodal distribution characterized by the presence of 14 large chromosomes (indicated by blue arrowheads) and 18 small chromosomes (denoted by yellow arrowheads). Chromosomes were counter-stained by DAPI. Scale bars indicate 10 µm

To explore the suitability of hairy roots and regenerants for cytogenetic studies and assess their chromosomal stability relative to the wild type, DAPI-stained mitotic chromosomes were analyzed (Fig. 5). In wild-type *A. axillaris* plants, 32 chromosomes were counted (2n = 32). The karyotype exhibited a distinct bimodal distribution that included 14 large and 18 small chromosomes (Fig. 5A). Both hairy roots (Line 7, Fig. 5B) and regenerants (Fig. 5C) had identical chromosome number and bimodal karyotypes. This highlights the potential utility of hairy root lines and their regenerant plants for cytogenetic studies.

## Discussion

The hairy root transformation system represents a versatile tool for plant biotechnology and may provide an option for species recalcitrant to transformation by *A. tumefaciens* [9, 16]. The two main methods for obtaining hairy root cultures are immersion-based and injection-based inoculation. The immersion method involves immersing the explants in a bacterial suspension. Infection time and duration of co-cultivation are critical factors to be tested for the plant species under study. The injection method requires direct injection of the bacterial suspension into the plant tissue using a sterile syringe. After a variable incubation period, depending on the plant species being studied, hairy roots emerge at wounding sites: the cutting site of explants (in the case of immersion-based method) or the site of injection. In general, both methods are effective in inducing hairy roots, although, in some species, the transformation efficiency by the injection method is higher than after dipping the explants [17, 18].

Hairy roots were previously induced in *A. thaliana* by immersing hypocotyl or leaf explants in a bacterial suspension [19–22]. The highest root induction rate (95%) was achieved by pre-culturing leaf explants on callus induction medium for four days and co-cultivation with the agrobacterial suspension for five minutes, followed by cultivation on standard medium [22]. Hairy roots were also induced by injecting an agrobacterial suspension into the middle of the rosette [23]. However, the efficiency of transformation was not reported. In our study, we tested the suitability of the inflorescence primary stem of *A. thaliana* for the injection-based method. The transformation efficiency reached 95% in one-month-old seedlings, comparable to experiments with leaf explants [22], but without the need to prepare explants and transfer them to different media. The suitability of the inflorescence stem for injection-based transformation was verified in *C. hirsuta*.

Regeneration of plant tissue, including hairy roots, is a significant bottleneck in plant biotechnology. It is a highly variable process that depends on species, ecotype, or cultivar. Therefore, optimizing regeneration conditions for each plant material is a necessary step. In our studies, we developed a regeneration protocol for hairy roots of several Brassicaceae species belonging to different supertribes (lineages) of this family (*A. thaliana* and *C. hirsuta* belong to the Camelinodae, *B. napus* belongs to the Brassicodae, and *A. axillaris* belongs to the Arabodae; [24]). We suggest that the protocol may also apply to establishing root cultures in other Brassicaceae species. If little or no regeneration is observed in other species or cultivars, the protocol will need to be adjusted. For example, a different cytokinin/auxin ratio (5 mg/L BAP and 5 mg/L NAA) successfully elicited shoot formation in various cultivars of *B. oleracea* [25, 26]. Alternatively, another type of cytokinin can be used, as shown when thidiazuron was used instead of BAP to regenerate hairy roots in *B. campestris* [26]. Higher cultivation temperature has also been shown to accelerate hormone-induced callus formation and shoot regeneration in *A. thaliana* [27]. Therefore, hairy roots can be cultivated at warmer temperatures during regeneration.

Using *A. axillaris* T0 regenerants, we tested the possibility of re-transformation, i.e., transforming a transgenic plant to insert a new trait. Regenerated plants containing *Ri*-derived T-DNAs from the first transformation were re-transformed with an *Agrobacterium* strain carrying a *Ri* plasmid and a binary vector encoding a VENUS fluorescent protein. Successful re-transformation was demonstrated by the detection of fluorescent signals in cell nuclei of hairy roots induced in T0 regenerants. Previous studies have used hairy roots for subcellular protein localization by detecting a fluorescent protein fused with the protein of interest (e.g., [28, 29]). Therefore, this double transformation protocol can be employed to detect protein localization in regenerating plants that have difficulty producing viable seeds.

The low seed germination rate is a limiting factor for studies of many endemic or rare species. Application of KNO_3_ and GA_3_ and exposure to low temperatures enhanced the release of seed dormancy in *A. axillaris*, consistent with studies in other species (e.g., [14, 30, 31]). Alternatively, seed dormancy can be overcome by embryo rescue. In this technique, immature plant embryos are excised and cultured on special media. ER has been used to rescue non-viable embryos, avoid secondary dormancy, and accelerate the transgenerational transition. Research studies using embryo rescue techniques to develop interspecific hybrids in *Brassica* species have shown that the effectiveness of this method depends on the female genotype, embryo developmental stage, and media composition (e.g., [32–34]). Accordingly, we developed an effective embryo rescue protocol for immature *A. axillaris* seeds using a medium containing the hormones IAA, BAP, and GA_3_. Similar to *B. napus* cv. DH12075 [12], embryos at the torpedo developmental stage seemed most suitable for ER in *A. axillaris*.

It has been reported that hairy root cultures are karyologically stable (e.g., [35–38]) and, therefore, can serve as a continuous source of root metaphase chromosomes for cytogenetic analyses. Hairy roots have been used to prepare intact chromosomes in a pea line with a reconstructed karyotype [39]. Such lines are often difficult to propagate due to the limited number of viable seeds and problems associated with generative propagation. In *A. axillaris*, we assessed the suitability of hairy roots and hairy root-derived regenerants for cytogenetic investigations. We found no significant chromosomal abnormalities compared with the wild-type plants. Therefore, hairy root cultures and regenerants can serve as a valuable source of mitotic and meiotic chromosomes in rare species, difficult-to-germinate species, and species with limited numbers of meristematic and sporogenous cells.

## Conclusions

In recent years, in vitro cultures have attracted considerable attention in the context of the conservation of endemic, rare, and endangered plant species (reviewed in [40, 41]). This interest stems from the inherent ability of biotechnologies to enable the rapid generation of new plants from limited quantities of parental tissue, thus avoiding the need for repeated collection of plant material from natural habitats. In our study, we successfully induced hairy root in vitro cultures and generated T0 regenerants from the rare crucifer species *Asperuginoides axillaris*. The plant material derived from both hairy roots and regenerants proved amenable to cytogenetic investigations. The inherent advantage of hairy roots, namely their ease of propagation on hormone-free media, also makes them an invaluable and sustainable source of plant material for various molecular biology and cytogenetic analyses.

## Methods

### Plant materials

Our study used *A. thaliana* ecotype Col-0 and *C. hirsuta* [42]. Seeds of *A. axillaris* were collected in Iran, Semnan Province, Damghan, by Hamid Moazzeni.

### Hairy root culture in *A. thaliana* and *C. hirsuta*

Seeds were sterilized by chlorine gas for six hours, followed by one hour of ventilation in sterile conditions, cold stratified at 4 °C for 24 h, and germinated in plant tissue culture boxes containing 50% Murashige and Skoog (MS) medium (Duchefa) with 5 g/L sucrose (Penta) for the indicated time (4 or 6 weeks for *A. thaliana*, 4 weeks for *C. hirsuta*) at 21 °C, 16-h light/8-h dark photoperiod and 150 μmol/m^2^/s light intensity. Hairy roots were obtained by transforming plants with the transconjugant *Ti*-less *A. tumefaciens* C58C1 carrying a hairy-root-inducing plasmid *pRiA4b* [43]. The agrobacterial suspension was grown for 18 h in Luria-Broth medium (10 g/L of tryptone [Duchefa], 5 g/L of yeast extract [Duchefa], 5 g/L of sodium chloride [Lachner], pH = 7.0) at 28 °C and injected with an insulin syringe into the basal part of the inflorescence stem. After 2–4 cultivation weeks, hairy roots emerging from the inoculation sites were excised and placed on a solid MS medium, including Gamborg B5 vitamins (MS + B5, Duchefa; 4.4 g/L), 0.3% phytagel (Sigma) and 30 g/L sucrose (Penta), supplemented with ticarcillin (500 mg/L, Duchefa) and cefotaxime (200 mg/L, Duchefa) to eliminate agrobacteria growth. Hairy roots were grown on Petri dishes at 24 °C in the dark and transferred to fresh MS + B5 media after 5–6 weeks of cultivation.

### Setting aseptic culture of wild-type *A. axillaris*

Wild-type *A. axillaris* seeds were surface-sterilized by several methods: (1) soaking in 70% ethanol for 5 min, (2) soaking in 70% ethanol for 2 min, followed by soaking in 0.5% sodium hypochlorite solution for 5 min (in both cases, seeds were then washed three times using sterilized water) or (3) sterilized by chlorine gas for six hours, followed by one hour of ventilation in sterile conditions. Seeds were then germinated on plates containing either germination medium 1 (GM1; 4.8 g/L MS [Duchefa], 10 g/L sucrose [Penta], 10 g/L plant agar [Duchefa], 0.25 mg/mL GA_3_ [Duchefa]) or GM2 (4.4 g/L MS + B5 [Duchefa], 30 g/L sucrose [Penta], 0.3% phytagel [Sigma], 0.4% activated charcoal [Sigma], 0.1 mg/mL GA_3_ [Duchefa]). The sterilization method and cultivation media are indicated in specific experiments in Additional File 1: Table S1. Sterilization by chlorine gas was ineffective in wild-type seeds, as they all got contaminated by microbial contamination. Germinating seeds were transferred to the plant tissue culture boxes containing 1/2 MS medium with 5 g/L sucrose and cultivated at 21 °C, 16 h light/8 h dark photoperiod, and 150 μmol/m^2^/s light intensity.

### Hairy root culture in *A. axillaris*

Two months after sowing the seeds, three aseptically grown plants of *A. axillaris* were subjected to hairy root transformation. The transformation process was as described for *A. thaliana* and *C. hirsuta* plantlets, except that the agrobacterial suspension was injected into the stem instead of the inflorescence stem. Fourteen hairy roots lines were established and grown on Petri dishes with solid MS + B5 media with ticarcillin (500 mg/L) and cefotaxime (200 mg/L) at 24 °C in the dark and transferred to fresh media every four weeks of cultivation.

### Shoot regeneration from hairy roots

Hairy roots of *A. thaliana* (10 lines)*, C. hirsuta* (10 lines)*,* and *A. axillaris* (14 lines) were transferred to MS + B5 solid media with 30 g/L sucrose supplemented with cytokinin 6-benzylaminopurine (BAP, 5 mg/L, Duchefa), and auxin 1-naphthaleneacetic acid (NAA, 8 mg/L, Duchefa). Roots were grown at 21 °C (*A. thaliana* and *C. hirsuta*) or 24 °C (*A. axillaris*) with a 16-h light/8-h dark photoperiod and 150 μmol/m^2^/s light intensity and transferred to fresh media every 3–4 weeks. The excised shoots were cultivated on a shoot elongation medium (SEM; MS + B5 with 20 g/L sucrose supplemented with 0.5 mg/L BAP and 0.03 mg/L GA_3_). After 3–4 weeks, they were transferred to the root induction medium (RIM; MS + B5 with 10 g/L sucrose and 0.5 mg/L indole-3-butyric acid, IBA [Duchefa]).

### Re-transformation of *A. axillaris* regenerants

Transformation of hairy root-derived regenerants growing on RIM was performed with *Agrobacterium* C58C1 harboring both *Ri* plasmid and a binary vector carrying a transgene for constitutive nuclear-targeted VENUS fluorescent protein expression (*p35S: NLS-VENUS*). The plasmid was prepared using Moclo cloning, as described previously [12]. Regenerants were injected into the stems, and after 1–2 months, the hairy roots emerged at the injection site.

Hairy roots growing on a selective agent (kanamycin, 15 mg/L, Duchefa) were screened for a fluorescence signal. Samples for confocal microscopy were prepared as described [44]. Briefly, root tips were fixed in 4% PFA (Merck) in PBS-T (1X PBS with 0.05% Triton X100 [Serva] pH 7.4) for one hour under vacuum conditions at 4 ºC and washed three times in PBS-T (pH 7.4) for one hour. Subsequently, they were transferred to a fresh ClearSee alpha solution (10% (w/v) xylitol [Sigma], 15% (w/v) sodium deoxycholate [TCI], 25% (w/v) urea [Merck], 50 mM sodium sulfite [Sigma] in water). After clearing for five days at room temperature, the roots were counter-stained with SCRI Renaissance 2200 (Renaissance Chemicals Ltd). Visualization was performed using an upright microscope Zeiss Axio Imager.Z2 with confocal unit LSM 700 equipped with filters DAPI (F-set 49), GFP (F-set 38), and ZEN black software for image processing.

### Germination test of hairy root-derived *A. axillaris* T1 seeds

Seeds were sterilized by chlorine gas for six hours, followed by one hour of ventilation in sterile conditions. Seeds were (1) treated by soaking in solution of GA_3_ (1 mg/mL) and KNO_3_ (0.5%, Lachner) on filter paper for 24 h, (2) treated by cold stratification at 4 °C in the dark on a solid medium GM3 (MS + B5 solid medium with 30 g/L sucrose, 3 g/L phytagel, 0.4% activated charcoal, 0.5 mg/L GA_3_, 0.1% Plant Preserve Mixture [PPM, Plant Cell Technology]) for 7 or 14 days, or (3) treated by a combination of cold stratification for 14 days and soaking in a solution containing GA_3_ (1 mg/mL) and KNO_3_ (0.5%) on filter paper for 24 h. The cold stratification in the latter experiment either preceded or followed soaking. After treatment, seeds were transferred to a fresh GM3 and moved to the cultivation chamber with 21 °C, 16 h light/8 h dark photoperiod, and 150 μmol/m^2^/s light intensity. Control seeds were sown directly onto GM3 and placed in the cultivation chamber. Three replicates with 15–30 seeds per replicate were used. Germination percentage was calculated four weeks after transfer to 21 °C. Arcsine-transformed data were subjected to analysis of variance (ANOVA), followed by the Duncan Multiple Range Test (p < 0.05).

### Embryo rescue of immature *A. axillaris* seeds

The seeds were collected in a sterile Petri dish and washed with 70% ethanol for 1 min, followed by two washing steps with sterile water. Seeds were divided into seven categories according to their size and color: stage 1–2.8 ± 0.2 mm in seed diameter; stage 2–3.9 ± 0.2 mm; stage 3–4.4 ± 0.2 mm; stage 4–5.1 ± 0.2 mm; stage 5–5.6 ± 0.2 mm; stage 6–6.5 ± 0.2 mm; stage 7–5.6 ± 0.2 mm. Seeds of stages 1–5 were green, and those of stages 6 and 7 were yellow. Testa was entirely removed by tweezers. To test the suitability of different seed stages for ER, the seeds were cultivated on embryo rescue medium 4 (ERM4) containing 0.5 mg/L GA_3_, 1 mg/L BAP, and 1 mg/L IAA [Duchefa]. The germination rate was calculated after two weeks.

For staging embryo development, seeds of stages 5 and 6 were cleared using a chloral hydrate solution (chloral hydrate [Sigma-Aldrich]/Glycerol/Water, 8/1/3 w/v/v) in a 2 mL tube. Samples were cleared for one month at 4 °C with gentle rotation. Visualization was performed using a ZEISS Axioscope.A1 equipped with DIC optics with an Axiocam 506 CCD camera.

Seeds of stage 2 were fixed in 4% PFA in PBS-T (pH 7.4) for one hour under vacuum conditions at 4 °C. Then, samples were washed three times in PBS-T (pH 7.4) for one hour and transferred to fresh ClearSee alpha for 21 days. After clearing, a SCRI Renaissance 2200 (Renaissance Chemicals Ltd) was used to mark the cell walls. Visualization was performed using an upright microscope Zeiss Axio Imager.Z2 with confocal unit LSM 700 equipped with a DAPI filter (F-set 49), and images were processed with the ZEN black software.

To test the hormonal effect on ER effectiveness, the seeds of stages 4–5 were cultivated on plates with one of the embryo rescue media (ERM). All media consist of MS + B5, 30 g/L sucrose, 0.4% activated charcoal, and 0.5 mg/L GA_3_. Further, they contained either 1 mg/L BAP (ERM2), 1 mg/L IAA (ERM3), or a combination of the two hormones (ERM4). The control medium contained no auxin and cytokinin (ERM1).

Germinated seeds were transferred to MS + B5 medium with 30 g/L sucrose without any hormones or supplemented with either 0.5 mg/L BAP and 0.03 mg/L GA_3_ (SEM) or 0.5 mg/L IBA (RIM). The survival rate was calculated two months after the transfer.

### Mitotic chromosome spreads

Actively growing young roots were collected from cultivated wild-type plants, established hairy root Line 7, or T0 regenerants of Line 7. The root tips were pre-treated with ice-cold water for 12 h, fixed in freshly prepared fixative (ethanol/acetic acid, 3/1, v/v) for 24 h at 4 °C, and stored at − 20 °C until further use. Selected root tips were rinsed in distilled water (twice for 5 min) and citrate buffer (10 mM sodium citrate [Duchefa], pH 4.8; twice for 5 min) and digested in 0.3% (w/v) cellulase, cytohelicase, and pectolyase (Sigma-Aldrich) in citrate buffer at 37 °C for 90 min. After digestion, individual root tips were dissected on a microscope slide in approximately 10 µL acetic acid and covered with a cover slip. The cell material was spread evenly using tapping, thumb pressing, and gentle flame-heating. Finally, the slide was quickly frozen in liquid nitrogen, and the cover slip was flicked off with a razor blade. Slides were fixed in ethanol/acetic acid (3/1, v/v) and air-dried. Chromosomes were counter-stained with 2 µg/mL DAPI in Vectashield (Vector Laboratories). Preparations were analyzed and photographed using a Zeiss Axioimager epifluorescence microscope and a CoolCube camera (MetaSystems).

### Supplementary Information

**Additional file 1: Table S1.** Effect of media composition and treatments for breaking seed dormancy of wild-type and hairy root-derived *A. axillaris* seeds. **Table S2.** Genotyping of hairy root lines, regenerants, and T1 plants of *A*. *axillaris*. **Figure S1.** Fourteen independent hairy root (HR) lines of *A. axillaris* were monitored for the insertion of left and right regions of the *Ri *T-DNA (TL and TR, respectively) into their genome. **Figure S2.** Hairy root-derived regenerants of *A*. *axillaris*.

## Data Availability

Not applicable.

## Abbreviations

B5: Gamborg B5 vitamins
BAP: 6-Benzylaminopurine
ER: Embryo rescue
ERM: Embryo rescue medium
GA3: Gibberellic acid
GM: Germination medium
IAA: Indole-3-acetic acid
MS: Murashige and Skoog
NAA: 1-Naphthaleneacetic acid
RGM: Regeneration medium
RIM: Root induction medium
Rol: Root oncogenic loci
SEM: Shoot elongation medium
TL: Left region of T-DNA
TR: Right region of T-DNA
