# Supplementary material for: Injection-based hairy root induction and plant regeneration techniques in Brassicaceae
Source: Plant Methods. 2024 Feb 17;20:29. doi: 10.1186/s13007-024-01150-1 (PMC10874044; doi:10.1186/s13007-024-01150-1)
Supplement: Supplementary file 1 — Additional file 1: Table S1. Effect of media composition and treatments for breaking seed dormancy of wild-type and hairy root-derived A. axillaris seeds. Table S2. Genotyping of hairy root lines, regenerants, and T1 plants of A. axillaris. Figure S1. Fourteen independent hairy root (HR) lines of A. axillaris were monitored for the insertion of left and right regions of the Ri T-DNA (TL and TR, respectively) into their genome. Figure S2. Hairy root-derived regenerants of A. axillaris. [file 13007_2024_1150_MOESM1_ESM.pdf]

Additional File

Injection-based hairy root induction and plant regeneration techniques in Brassicaceae

Supplementary Table S1. Effect of media composition and treatments for breaking seed dormancy of wild-type and hairy root-derived *A. axillaris* seeds

Supplementary Table S2. Genotyping of hairy root lines, regenerants, and T1 plants of *A. axillaris*

Supplementary Figure S1. Fourteen independent hairy root lines of *A. axillaris* were monitored for the insertion of left and right regions of the *Ri* T-DNA (TL and TR, respectively) into their genome

Supplementary Figure S2. Hairy root-derived regenerants of *A. axillaris*

Supplementary Table S1. Effect of media composition and treatments for breaking seed dormancy of wild-type and hairy root-derived *A. axillaris* seeds. Analysis of the effect of cold stratification (moist chilling at 4 °C) and application of 1 mg/mL GA_3 and 0.5 % KNO_3 solution on seed germination was performed on germination medium 3 (GM3). After the indicated incubation time, seeds were transferred to 21 °C on fresh GM3. Germination percentage was calculated after one month of cultivation at 21 °C. [a, b, c, d], statistical analysis from three biological replicates with 15 – 30 seeds per replicate. Arcsine-transformed data were subjected to analysis of variance (ANOVA). The differences between the means were compared using the Duncan Multiple Range test ($p < 0.05$). Data from the germination test using GM3 are expressed as mean values ± SEM.

A. axillaris seeds	sterilization	treatment	cultivation	germination rate
wild-type	70 % EtOH	-	GM1	3.3 % (4/120)
	70 % EtOH, 0.5 % NaOCl	-	GM2	4.6 % (3/65)
	gas chlorine	-	GM2	0 % (0/20)
HR-derived T1	70 % EtOH, 0.5 % NaOCl	-	GM2	4.4 % (2/45)
	-	-	soil	2.5 % (2/80)
	gas chlorine	-	GM3	4.2 ± 0.2 % [ab]
	gas chlorine	7 days moist chilling	GM3	2.2 ± 2.2 % [a]
	gas chlorine	14 days moist chilling	GM3	7.2 ± 1.4 % [bc]
	gas chlorine	24 h imbibition in GA_3 + KNO_3	GM3	16.9 ± 1.3 % [c]
	gas chlorine	24 h imbibition in GA_3 + KNO_3, then 14 days moist chilling	GM3	35.6 ± 5.8 % [d]
	gas chlorine	14 days moist chilling, then 24 h imbibition in GA_3 + KNO_3	GM3	34.5 ± 6.1 % [d]

Composition of media:
GM1: 4.8 g/L Murashige & Skoog basal salt mixture (MS), 10 g/L sucrose, 10 g/L plant agar, 0.25 mg/mL GA_3
GM2: 4.4 g/L MS supplemented with B5 vitamins, 30 g/L sucrose, 3 g/L phytagel, 0.4 % charcoal, 0.1 mg/mL GA_3
GM3: 4.4 g/L MS supplemented with B5 vitamins, 30 g/L sucrose, 3 g/L phytagel, 0.4 % charcoal, 0.5 mg/L GA_3, 0.1 % Plant Preserve Mixture (PPM, Plant Cell Technology)

Supplementary Table S2. Genotyping of hairy root lines, regenerants, and T1 plants of *A. axillaris*. In all hairy root (HR) lines and derived regenerants (Reg), the left and right regions of the *Ri* T-DNA (TL and TR, respectively) were detected. Numbers in brackets refer to the number of positive plants from all tested plants. ER, the embryo rescue technique was successful in three lines. n.a., not available (Line 13 did not develop viable seeds).

HR Line	TL (HR)	TR (HR)	TL (Reg)	TR (Reg)	ER	TL (T1 plants)	TR (T1 plants)
Line 1	+	+					
Line 2	+	+	+ (4/4)	+ (4/4)	+	+ (8/8)	+ (7/8)
Line 3	+	+					
Line 4	+	+					
Line 5	+	+	+ (3/3)	+ (3/3)	+	+ (6/6)	+ (4/6)
Line 6	+	+					
Line 7	+	+	+ (5/5)	+ (5/5)	+	+ (7/7)	+ (7/7)
Line 8	+	+					
Line 9	+	+					
Line 10	+	+					
Line 11	+	+					
Line 12	+	+					
Line 13	+	+	+ (3/3)	+ (3/3)	n.a.	n.a.	n.a.
Line 14	+	+					

.

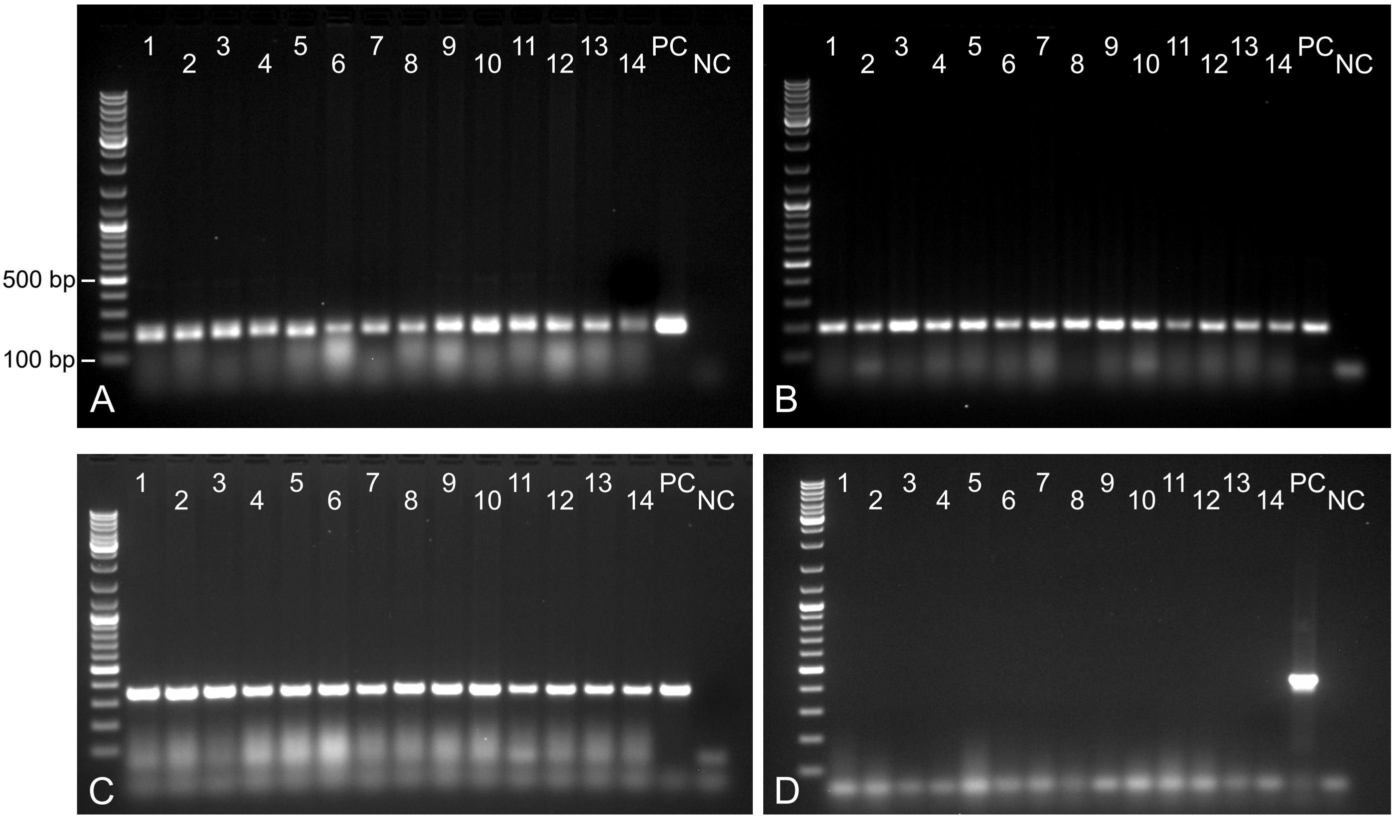

Supplementary Figure S1. Fourteen independent hairy root lines of *A. axillaris* were monitored for the insertion of left and right regions of the *Ri* T-DNA (TL and TR, respectively) into their genome. Genomic DNA extracted from hairy roots was subjected to PCR analysis using primers for TL-specific *rolA* (**A**), and TR-specific *aux1* (**B**) and *ags1* (**C**). The absence of *Agrobacterium* contamination was confirmed by the absence of the *virC* locus in extracted DNA (**D**). PC, positive control (plasmid *pRiA4b*). NC, negative control of PCR (no template included). Primers specific to each locus are listed in Jedličková et al., 2022 [12].

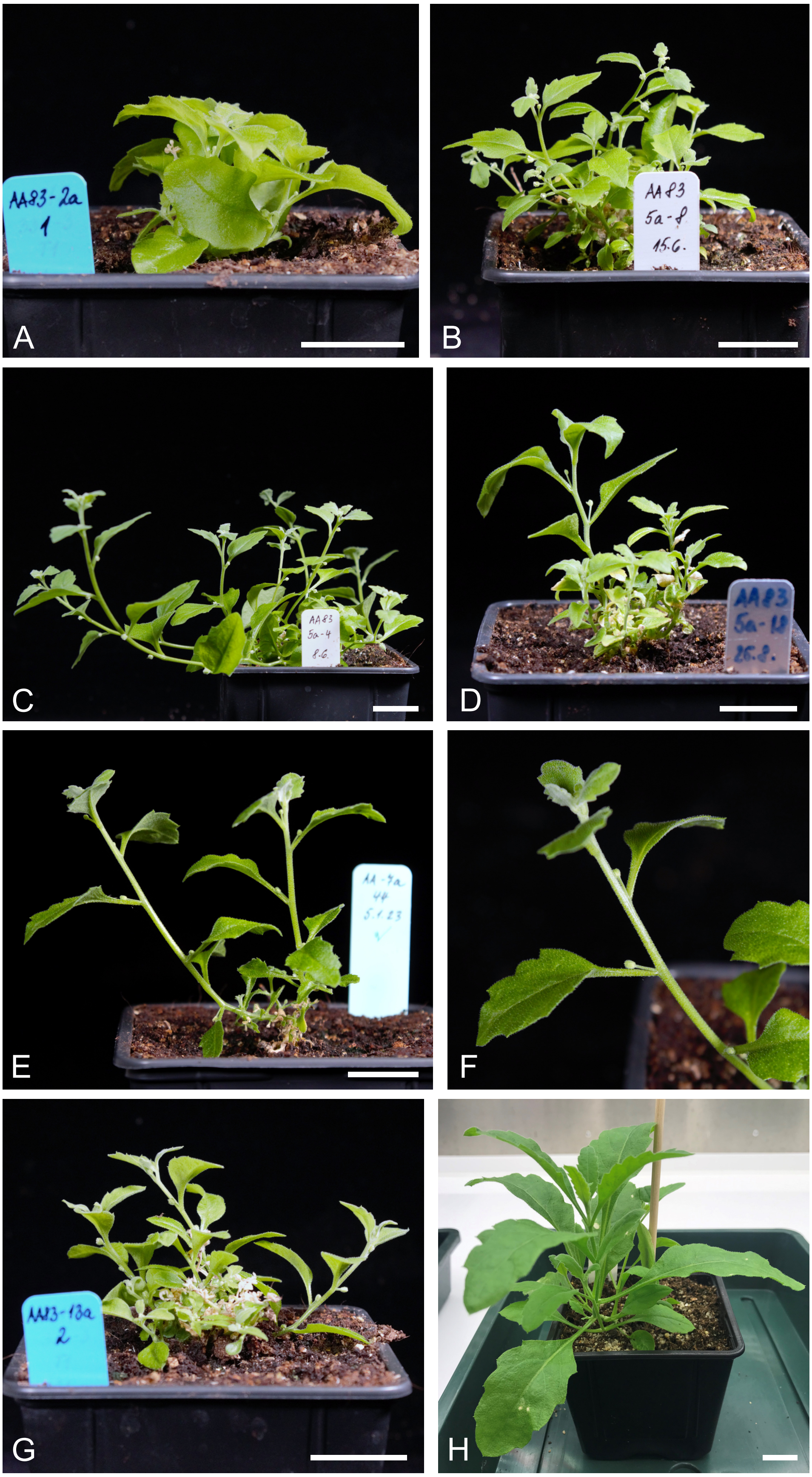

Supplementary Figure S2. Hairy root-derived regenerants (T0 plants) of *A. axillaris*. Representative pictures of regenerants from hairy root Line 2 (**A**), Line 5 (**B** – **D**), Line 7 (**E**, with detail of flower buds in **F**), and Line 13 (**G**). A wild-type plant is shown in (**H**) for comparison. Scale bars represent 2 cm.
